# Comparability and Stability of Serum Creatinine Concentration in Capillary and Venous Blood

**DOI:** 10.3389/bjbs.2023.11402

**Published:** 2023-09-11

**Authors:** Timothy Woolley, Emma Rutter, Macarena Staudenmaier

**Affiliations:** ^1^ Inuvi Diagnostics, Gloucestershire, United Kingdom; ^2^ Thriva Co., London, United Kingdom

**Keywords:** capillary blood, creatinine, sample stability, CKD, comparative analysis

## Abstract

**Background:** The use of self-collected capillary blood has several advantages over phlebotomy, as such finger-prick testing is rapidly becoming accepted as a routine sample type for adults. However, there is limited evidence that venous and capillary serum is comparable for many analytes. This study aimed to determine whether capillary samples could offer an alternative sampling method to venous samples for the assessment of serum creatinine using the enzymatic method and if this analyte was stable in unspun capillary blood for 3 days.

**Methods:** Matched capillary and venous blood samples were collected from 48 patients for the determination of serum creatinine, one set being processed on day zero, the other set being stored at ambient temperature and then processed on day three. Self-collected capillary blood was compared with phlebotomist-collected venous samples.

**Results:** Serum creatinine concentrations from venous and capillary blood samples taken on day zero were compared to concentrations in capillary blood from day three. Data produced showed serum creatinine concentrations from capillary and venous serum to be comparable.

**Conclusion:** It is believed that this is the first published study to determine if self-collected capillary blood sampling is an acceptable alternative to venous sampling for the measurement of serum creatinine concentration; our data indicates that there is no significant difference in results from unspun venous and capillary blood stored at room temperature for at least 3 days compared to venous blood tested on the same day of collection.

## Background

Chronic kidney disease (CKD) is a significant health issue affecting up to 10% of the UK adult population [1, 2]. With the prevalence of CKD increasing, the need for good screening methods to identify CKD risk is more important than ever. Likewise reduced access to health services, due in the main to the COVID-19 pandemic, has led to a concerning backlog of primary and secondary care appointments, therefore novel testing and monitoring processes that allow for remote testing could help address some of these growing issues.

Monitoring of glomerular filtration rate (GFR) is essential for the diagnosis and management of patients with CKD [3]. Although direct reference measurement methods using specific markers such as inulin are considered the gold standard, access is not readily available, as such GFR is usually estimated (eGFR) using serum creatinine concentration in conjunction with estimation formula, e.g., CKD-EPI calculation.

The New Opportunities for Early Renal Intervention by Computerised Assessment (NEOERICA) project found that although 10% of the UK population suffers from CKD, only 1.6% of patients had a recorded renal diagnosis [1]. These patients require regular monitoring, as such an accurate estimation of GFR is essential [4, 5], however, monitoring is resource heavy both in terms of patient commitment, access to phlebotomy, and laboratory services.

Point of Care (POC) methodology used within a hospital setting can be as accurate as laboratory analysis for the assessment of eGFR [6], yet POC methodology is expensive when compared to laboratory options, requiring either the placement of a dedicated analyser with consumables or health professional oversight, in addition, POC analysis in whole blood remains challenging due to the complexity of its matrix [7].

Regular outpatient visits are time-consuming, inconvenient, and expensive in terms of clinic setup, staffing, and the time required by the patient. Although ready access to state-of-the-art laboratory services offset these issues, the need for person-to-person contact is inevitable, and post Covid access to primary and secondary care services has suffered. Therefore, easy access to state-of-the-art laboratory assays that do not require either a phlebotomist or health professional in attendance could reduce costs and non-attendance rates while improving patient care and satisfaction. Likewise, self-collection methods do not require in-person attendance at primary care centers, and as such this may allow for the earlier detection and easy monitoring of CKD.

Recent advances in technology have put novel collection methods using limited amounts of blood within the grasp of many laboratories, e.g., dried blood spot (DBS) [8]. Dried blood spots are easy to collect, can be done by untrained users, consumables are cheap, and analytes are stable for protracted periods at ambient temperatures. However, the equipment to perform analyte analysis on DBS are expensive, requires specialist personnel, and as such is not always found in every laboratory. However, Capillary Blood Testing (CBT) is an ideal alternative. Capillary blood is easy to obtain with minimal direction, can be performed at home, uses fewer resources, and therefore creates less waste [9, 10]. In addition, serum obtained from capillary blood can easily be used on standard laboratory analysers using the same methodology as venous serum and as such does not require additional equipment and fits into the routine laboratory sample pathway.

Recently published evidence from CBT suggests that serum creatinine concentrations appear significantly different from venous blood [10], however, this study used the Jaffe creatinine method which is known to overestimate creatinine concentration in certain circumstances [10], including delays in sample separation [10, 11].

Drion et al. (2012) found that the Jaffe method overestimated serum creatinine concentration by 21%, 12%, 10% for target values of 52, 73, and 94 μmol/L, respectively. Whereas in the enzymatic creatinine method, these values were 0%, −1%, −2%, respectively. This led to the Jaffe derived eGFR staging patients into a lower CKD category. Downgrading to a lower CKD stage occurred in 1%–42%, 2%–37%, and 12%–78.9% of patients for the 10th and 90th percentile respectively in CKD categories 45–60, 60–90, and >90 mL/min/1.73 m^2^. Using enzymatic techniques, downgrading occurred only in 2%–4% of patients [11].

The manufacturer’s instruction for use note that creatinine is stable for 7 days in serum and plasma when stored between 2°C and 25°C, and there are several studies supporting the stability of creatinine in whole blood venous samples for 48 h using enzymatic methods [12–14], however, there are no studies comparing enzymatic creatinine comparability and stability in capillary and venous samples over protracted timescales and at different temperatures that could be present during sample postage.

This study aimed to establish the validity of measuring serum creatinine concentrations using an enzymatic method in capillary blood as a viable alternative to venous blood using two sets of samples, one pair (venous and capillary) being collected and processed on day zero, while the second pair (venous and capillary) would be collected on day zero, stored at ambient temperature and processed on day three. Additional samples were also collected and put through three different temperatures, these were 4°C for 24 h, ambient for 24 h, and 37°C for 24 h. This second set of samples being used to mimic samples being sent via the postal network.

## Materials and Methods

Since this was a feasibility study, no comprehensive power analysis was performed. Sample size was based on our standard clinical chemistry ISO15189 assay verification protocols. This study included forty-nine patients requiring creatinine testing providing both two venous and two capillary samples. Once consent was given, all participants were provided with a standard collection guide, and samples were collected without further direction, collection was performed immediately after the venous samples. Venous samples were collected using a trained phlebotomist.

Venous samples were collected using Becton Dickinson (Oxford, United Kingdom) Serum Separator Tubes (SST), while capillary samples were collected using Becton Dickinson Microtainer SST tubes and lancets. One pair of venous and capillary samples were then centrifuged together at 2000 g for 10 min within 6 h of collection and analysed together on the same day using the Beckman Coulter (Oxford Hill, United Kingdom) enzymatic Creatinine assay on a DXC 700 AU chemistry platform. The second set of samples were stored at ambient temperature (16°C–22°C) for 3 days before centrifugation and again tested together on the same platform. An additional set of samples (*n* = 5) were also taken, one sample being tested on day 1, and the second being tested on day 3, this second set of samples underwent a storage cycle over three temperatures, these were 24 h at 2°C–8°C, 24 h at ambient (16°C–22°C) and 24 h at 37°C.

## Statistical Analysis

Pearson’s Correlation and Bland-Altman analysis was used to assess the strength of the relationship and level of agreement between creatinine concentrations in venous and capillary blood for samples taken on day zero and between venous serum from day zero and capillary serum from day three as described by Altman et al. and Thienpont et al. [14, 15]. All statistical analysis was performed using Analyse-It method validation edition (Analyse-It, Leeds, United Kingdom). Acceptance criteria were a correlation score of r > 0.95, and a Bland-Altman result within the Total Allowable Error for Creatinine. The analytical within-subject coefficient of variation of Creatinine is 4.5% (95% CI: 4.1%–5.7%) and Total Allowable Error is quoted as being 8%–10% [16].

## Results

49 paired samples (two capillary and two venous) were obtained from generally well people. Table 1 shows the raw data produced from these samples, alongside a percentage difference between the venous day zero and the capillary day three (*r* = 0.9737).

**TABLE 1:** Day 0 and Day 3—creatinine measurements in venous and capillary serum (units μmol/L, *n* = 49).

Patient	Venous Day 0	Capillary Day 0	Venous Day 3	Capillary Day 3	% Difference Day 0 Venous v Day 3 Capillary
1	63	63	60	64	−1.6
2	80	76	81	75	6.3
3	64	66	67	70	−9.4
4	60	61	61	61	−1.7
5	65	64	65	65	0.0
6	61	62	63	63	−3.3
7	59	56	59	58	1.7
8	85	86	86	80	5.9
9	62	61	65	62	0.0
10	59	58	58	60	−1.7
11	69	66	67	65	5.8
12	62	61	61	64	−3.2
13	65	62	65	62	4.6
14	73	70	73	71	2.7
15	63	60	62	59	6.3
16	96	89	91	91	5.2
17	59	55	58	59	0.0
18	96	89	93	87	9.4
19	86	80	87	87	−1.2
20	67	63	64	64	4.5
21	71	69	70	67	5.6
22	53	52	52	51	3.8
23	98	100	99	100	−2.0
24	64	63	61	61	4.7
25	117	116	113	116	0.9
26	76	80	72	79	−3.9
27	66	60	60	61	7.6
28	79	81	77	78	1.3
29	63	63	60	64	−1.6
30	80	76	81	75	6.3
31	76	73	76	74	2.6
32	64	66	67	70	−9.4
33	60	61	61	61	−1.7
34	65	64	65	65	0.0
35	59	56	59	58	1.7
36	85	86	86	80	5.9
37	62	61	65	62	0.0
38	87	88	81	82	5.7
39	89	89	92	87	2.2
40	94	93	92	90	4.3
41	87	81	89	86	1.1
42	74	72	80	75	−1.4
43	87	89	92	89	−2.3
44	88	85	87	86	2.3
45	59	57	60	57	3.4
46	70	68	70	68	2.9
47	103	97	97	98	4.9
48	85	81	89	79	7.1

Correlation between venous serum on day one and capillary serum from day three is *r* = 0.9737.

**FIGURE 1:**
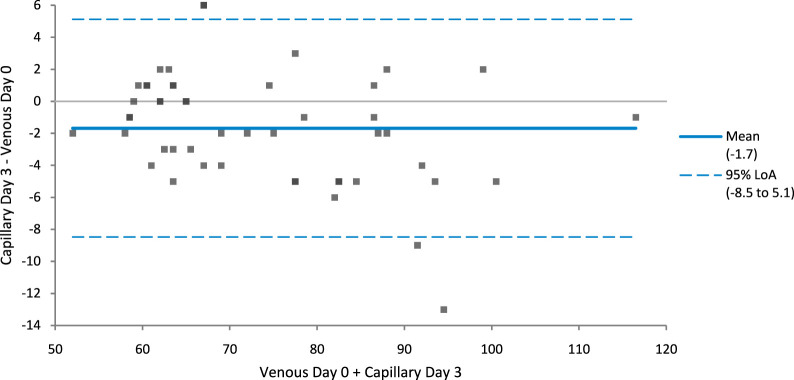
Bland-Altman plot comparing creatinine concentration from venous samples tested on day zero and capillary samples tested on day three indicates a negative mean bias of 1.7 μmol/L. Limits of agreement are shown in blue dotted lines, line of identity is shown as solid blue line.

A Bland Altman plot (Figure 1) comparing creatinine concentration from venous samples tested on day zero and capillary samples tested on day three indicates a negative mean bias of 1.7 μmol/L.

Additional samples were then obtained to assess creatinine stability over 3 days in unseparated blood using an extended temperature cycle, this included 24 h at 2°C–8°C, 24 h at ambient (16°C–22°C) and 24 h 37°C (Table 2). Correlation between capillary serum tested on day one and day three is *r* = 1.0.

**TABLE 2:** Day 1 and Day 3—creatinine measurements in capillary serum after an extended temperature cycle, samples were stored unseparated for 3 days, the temperatures included 24 h at 2°C–8°C, 24 h at ambient (16°C–22°C) and 24 h 37°C (units μmol/L, *n* = 5).

Patient	Day 1	Day 3
1	59	60
2	95	96
3	51	52
4	84	84
5	74	75

Correlation between capillary serum tested on day one and serum from day three is *r* = 1.0.

## Discussion

The use of serum creatinine measurement alongside eGFR to diagnose and monitor kidney function is well established. CBT, therefore, presents some advantages over traditional venous sampling, especially given capillary sample collection does not require attendance at primary or secondary care facilities. Frequent primary and secondary care visits for patient monitoring have a significant impact both on the patient and the healthcare provider. However, to fully utilize self-collected capillary sampling it is imperative not only to confirm comparability between venous and capillary samples but also to ensure analyte stability over routine transport times and temperatures.

Several studies have shown capillary sampling to be preferred to venous collection [9, 17, 18], in addition, this simpler and less-invasive technique also requires less raw materials and therefore produces less waste. The self-collection process is easy to follow and can be performed as and when the patient is ready, therefore the sample may better represent a “resting state” specimen. Self-collection capillary sampling utilizing centralized laboratory analysis gives patients more control while also ensuring very high-quality laboratory analysis. Self-collection followed by sample postage would vastly reduce waiting times and all but removes the need for patients to visit primary and secondary care for blood collection, which would inevitably reduce carbon emissions and traffic congestion around healthcare facilities as well as none attendance rates.

Although there are numerous papers validating the use of capillary blood for infectious disease testing [18–20], at present there is a limited but growing volume of data on the comparability and stability of biochemical serum analytes in capillary and venous blood [9, 21, 22]. This study is the first to compare capillary and venous samples for the determination of serum creatinine concentration using the enzymatic creatinine method, whilst also providing whole blood stability data over an extended temperature range.

The above findings show that there is very good agreement between capillary and venous serum creatinine concentrations, both on day zero and on day three at ambient temperatures over 3 days. Bland-Altman analysis identified no clinically significant bias between these matrices either on day zero or day three, with the Bland-Altman analysis showing a negative bias of 1.7 μmol/L. The majority of storage data was produced from samples stored at ambient temperatures, of the 49 sample sets, 10 day 3 capillary samples compared to day 0 venous samples had percentage differences over the within-subject coefficient of variation limit of 5.6% but only three capillary samples breached the Total Allowable Error limit of 8% when comparing capillary samples on day three to venous samples tested on day 0.

An additional five samples were put through a rigorous extended temperature cycle that moved from 24 h at 2°C–8°C, 24 h at ambient (16°C–22°C), and finally 24 h at 37°C. Although sample numbers tested were limited, and the creatinine concentrations were from healthy individuals the data presented suggests samples are stable unseparated for up to 3 days over a wide temperature range, and therefore creatinine concentrations tested using the enzymatic method could be assessed using at-home self-collected capillary samples transported to the laboratory via a standard postal/courier network.

Although both venipuncture and capillary sampling were performed in a controlled setting for experimental purposes, in practice CBT would be performed at home and be delivered to the laboratory through the postal service. We therefore simulated a standard delivery time of 3 days, the study did focus on storage at ambient temperatures, however additional, albeit limited work was done to cover a much wider temperature range.

These results could be of particular importance as health services recover from the COVID-19 pandemic, allowing the use of remote blood testing for both diagnostic and monitoring purposes, freeing up phlebotomy and outpatient resource while still utilizing central laboratory services, thus feeding results directly into standard patient care pathways.

It is however critical that comparisons between blood matrices are included as part of the laboratories verification process for each analyte offered as some may behave differently between capillary and venous blood, likewise, sample stability must also be evaluated. It is noted that this is a feasibility study only, does not include very low or very high creatinine concentrations, and likewise did not cover whole blood stability over more than 72 h.

## Conclusion

This work supports the use of CBT for the determination of creatinine concentrations using the enzymatic method as an alternative to venous sampling. This feasibility study provides promising data for the long-term stability of whole blood capillary samples over a wide range of temperatures and therefore opens the possibility of using CBT to support the diagnosis and monitoring of CKD via self-collection.

## Summary Table

### What Is Known About This Subject

• 10% of the UK population suffers from CKD but only 1.6% of patients have a recorded renal diagnosis. These patients require regular monitoring.
• An accurate estimation of GFR is essential. Serum creatinine measurement using venous sampling alongside eGFR is the most common method.
• Phlebotomy is resource heavy. Home-based blood sampling could be beneficial in terms of cost and patients’ wellbeing.

### What This Work Adds

• This is the first study to compare venous and self-collected capillary blood sampling for the measurement of serum creatinine.
• Creatinine stability in unseparated blood over a range of temperatures for up to 3 days was also tested.
• Home based self-collected capillary sampling for the measurement of serum creatinine is a viable alternative to phlebotomy.

This work represents an advance in biomedical science because is confirms that capillary blood is a viable alternative to venous blood for the measurement of serum creatinine using the enzymatic method.

## Data Availability

The original contributions presented in the study are included in the article/supplementary material, further inquiries can be directed to the corresponding author.

## Abbreviations

CKD, chronic kidney disease; GFR, glomerular filtration rate; eGFR, estimated glomerular filtration rate; POC, point of care; DBS, dried blood spot; CBT, capillary blood testing.
